# Caspase-10 Is the Key Initiator Caspase Involved in Tributyltin-Mediated Apoptosis in Human Immune Cells

**DOI:** 10.1155/2012/395482

**Published:** 2012-01-12

**Authors:** Harald F. Krug

**Affiliations:** Department Materials Meet Life, Empa Swiss Federal Laboratories for Materials Science and Technology, Lerchenfeldstrasse 5, 9014 St. Gallen, Switzerland

## Abstract

Tributyltin (TBT) is one of the most toxic compounds produced by man and distributed in the environment. A multitude of toxic activities have been described, for example, immunotoxic, neurotoxic, and endocrine disruptive effects. Moreover, it has been shown for many cell types that they undergo apoptosis after treatment with TBT and the cell death of immune cells could be the molecular background of its immunotoxic effect. As low as 200 nM up to 1 **μ**M of TBT induces all signs of apoptosis in Jurkat T cells within 1 to 24 hrs of treatment. When compared to Fas-ligand control stimulation, the same sequence of events occurs: membrane blebbing, phosphatidylserine externalisation, the activation of the “death-inducing signalling complex,” and the following sequence of cleavage processes. In genetically modified caspase-8-deficient Jurkat cells, the apoptotic effects are only slightly reduced, whereas, in FADD-negative Jurkat cells, the TBT effect is significantly diminished. We could show that caspase-10 is recruited by the TRAIL-R2 receptor and apoptosis is totally prevented when caspase-10 is specifically inhibited in all three cell lines.

## 1. Introduction

Tributyltin (TBT) is one of the most toxic compounds still used in antifouling paints for large commercial ships thereby distributed within the aquatic environment. Its distribution and accumulation in aquatic organisms leads to severe effects and has already reduced the number of snail species in the near of sea lanes and harbours [1]. Moreover, the trophic transfer has been demonstrated [2], and the accumulation within the food chain up to the level of marine mammals has reached concentrations that might be biological relevant [3–6]. The most prominent biological effect investigated so far is the so-called imposex within sea snails and dogwhelks [1, 7, 8], and this mechanism is used as biomonitoring tool for organotin compounds [9]. Despite the fact that a lot of studies have been carried out, the underlying molecular mechanism remains unclear [10, 11]. It has been proposed that the inhibition of aromatase activity alters the ratio of the hormones inducing the development of imposex, the imposition of male sex characteristics on female snails [1, 12], but other studies came to other results [11, 13].

As organotin compounds were still used and accumulate in the environment as well as in the food chain, the exposure of mammals and humans increases steadily. Moreover, it has been described earlier that organotin compounds, especially TBT, have a clear immunotoxic effect in mammals [14–16], and this might be due to their exorbitant induction of apoptosis [17, 18]. The effective concentration of TBT to induce apoptosis in the majority of treated cells is around or below 1 *μ*M and has been shown *in vitro* [14, 18–27] as well as *in vivo* [28, 29]. The question whether the disturbance of the intracellular calcium homeostasis is responsible for the onset of apoptosis [22, 27, 30–34] or the direct effect on mitochondrial functions is the first event [18, 20, 24, 32, 33, 35] is under discussion for a long time. Stridh et al. [18, 23] have shown a decade ago that TBT induces apoptosis via the activation of caspases in various human cells, the link for this caspase activation was not yet found. The most obvious players have been discussed to be the increase in calcium concentration or the opening of the permeability pore of the mitochondria. But induction of apoptosis has been demonstrated for very low concentrations of TBT which do not induce calcium influx [27], and caspases are often inhibited by high calcium concentrations [23]. Some years ago, evidence arose that mitochondria-independent mechanisms contribute to the induction of apoptosis and possibly death receptors or direct caspase activation are involved in the TBT induced effect [36–39].

It is now generally accepted that the programmed cell death can be physiologically induced via death receptors on the surface of the cells, activated by specific ligands that are strictly controlled for instance during development or inflammation [40] leading to the formation of the so-called “death-inducing signalling complex” or DISC [41]. Moreover, it has been shown that at least lymphoid cells can be discriminated into type I and type II cells and only type II cells are strongly dependent on functional mitochondria for their apoptotic machinery [42]. Jurkat T-lymphoblastoid cells are type II cells and present a special tool for the investigation of mitochondrial-dependent cell death characteristics. Additionally, genetic modifications of the DISC within these cells enable a closer look at which point the sequence of events is started after TBT-treatment.

In the present study, the mechanism of TBT-induced apoptosis has been investigated by the use of Jurkat T-cells and two variants, caspase-8 and FADD-deficient Jurkat cells, that provide a direct insight into the death-receptor-coupled mechanisms. The data presented here point to the involvement of initiator caspase activation, especially from caspase-10, and are discussed in terms of the potential immunotoxic role of TBT in exposed mammals.

## 2. Results

### 2.1. TBT Induces Apoptosis in Human Jurkat Cells

**Figure 1:**
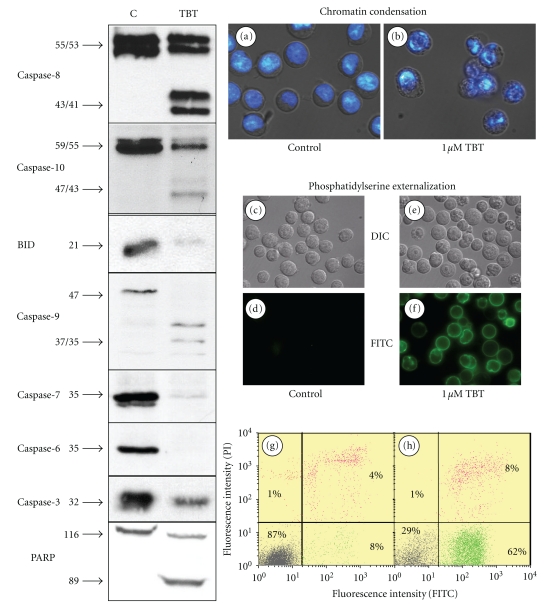
Induction of apoptosis in Jurkat T cells by TBT. Jurkat cells were treated with 1 *μ*M TBT or ethanol (control) for 4 h before samples were analysed. Nuclei of vehicle-treated control cells (a) and after TBT treatment (b) were stained with Hoechst 33342 and analysed with a fluorescence microscope. Both pictures show an overlay of differential interference contrast light microscopy pictures with the fluorescence pictures. From the same experiment, samples were stained with annexin V-FITC for phosphatidylserine externalisation (c–h). Control cells were shown with DIC contrast (c), and no green fluorescence could be detected at 525 ± 12.5 nm (d). TBT-treated cells exhibit ruffled membranes and granular cytoplasm (e) and strong PS-labelling at the plasma membrane (f). The annexin-positive cells were further quantified by flow cytometry. The quadrant analysis of double labelled cells is shown for control (g) and TBT-treated cells (h). 10 000 cells of each sample were counted, and the percentage of viable cells (lower left quadrant, grey dots), apoptotic cells (lower right, green dots), and necrotic cells (upper two quadrants, red dots) were given in the dot blots. On the left side of the figure, immunoblots for 8 different proteins were shown. Protein names and molecular weights are given aside the blots. Left lane: control sample; right lane: TBT-treated sample.

When human immune cells were treated with TBT, changes of morphological as well as biochemical parameters of apoptosis can be observed. In all experiments done in this study, we used 1 *μ*M TBT, a concentration that induces apoptosis in the majority of the treated cells within 4 hours. After that, time membrane blebbing and the externalisation of phosphatidylserine (PS) occur and chromatin condensation could be observed in Jurkat A3 T-cells (Figures 1(a) and 1(b)). Chromatin condensation was shown by the use of the DNA dye Hoechst 33342, and PS on the outer leaflet of the plasma membrane is detected with Annexin V-FITC by flow cytometry (FACS) and fluorescence microscopy (Figures 1(c)–1(h)). As demonstrated by two typical FACS dot blots, more than 60% of the treated cells undergo apoptosis and exhibit green fluorescence at the plasma membrane without being necrotic as the counter staining with propidium iodide demonstrates clearly. Looking closer to the different proteins that are involved in the apoptotic machinery, the complete sequence of events from initiator caspases down to death substrates is switched on. Focussing at the level of initiator caspases, both, caspase-8 and caspase-10, are cleaved and their active subunits can be detected by western blotting (Figure 1, left). Downstream the initiator caspases, the BID protein is an important linker to the mitochondrial pathway in type II cells and this protein is cleaved after TBT treatment. From the multitude of caspases downstream of the mitochondria we tested for procaspase-9, -7, -6, and -3 and found all these proteases cleaved. As one of the most prominent death substrates poly(ADP-ribose) polymerase (PARP) has also been shown to be cleaved within this series of events (Figure 1, left). Moreover, we tested hepatocytes transfected with a fusion protein of cytochrome c/green fluorescent protein (kindly provided by D. Green, La Jolla Institute for Allergy and Immunology, San Diego, USA) and found the release of cytochrome c after TBT treatment (data not shown).

### 2.2. Apoptosis Is Diminished in Deficient Cell Lines and Dependent from Caspase Activity

**Figure 2:**
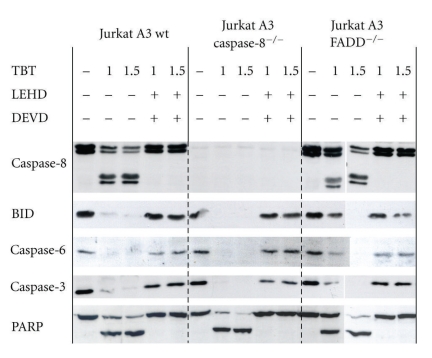
Inhibition of TBT-induced apoptosis by caspase-9 and caspase-3 inhibitors. Western blots of 5 different proteins in Jurkat A3 parental and the two deficient cell lines are shown. Cells were treated with 1 or 1.5 *μ*M TBT for 4 h before proteins were separated on SDS-gels and immunoblotted. Another set of samples was preincubated for 1 h with the inhibitors for caspase-3 (DEVD, 10 *μ*M) and caspase-9 (LEHD, 30 *μ*M) before TBT (1 *μ*M or 1.5 *μ*M) was added. For molecular weights of proteins and cleavage products, compare Figure 1.

**Figure 3:**
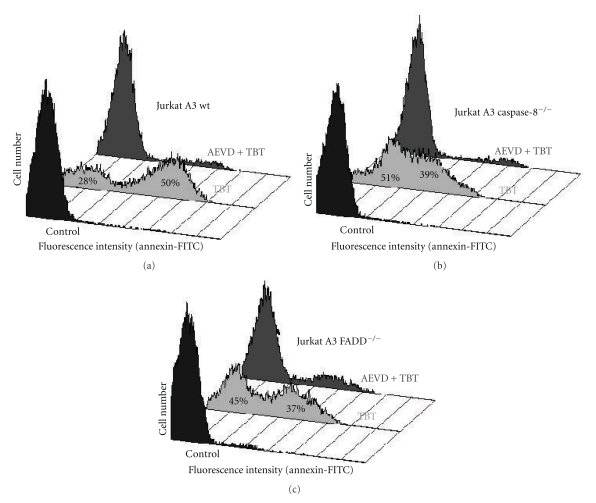
Caspase-10 inhibition prevents TBT-induced apoptosis in all variants of Jurkat A3 cells. Jurkat cells were pretreated with the caspase-10 inhibitor AEVD (8.7 *μ*M, 1 h) before TBT was added (1 *μ*M, 4 h). Then, the cells were stained with annexin V-FITC/PI to separate apoptotic cells from necrotic and viable cells by flow cytometry. The histograms show the fluorescence intensity of PI-negative only (compare lower two quadrants in Figures 1(g) and 1(h)). Numbers given for TBT-treated samples represent the percentage of total cells counted (10 000). Presented are the histograms of all three Jurkat A3 variants.

**Figure 4:**
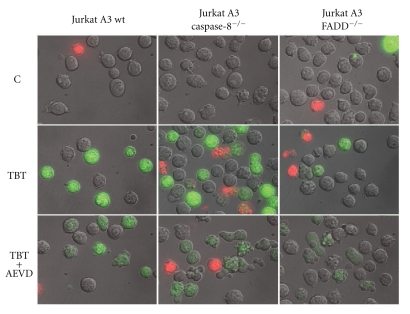
Caspase activity induced by TBT treatment in Jurkat A3 cells. Jurkat cells were pretreated with the caspase-10 inhibitor AEVD (8.7 *μ*M, 1 h) before TBT was added (1 *μ*M, 4 h). At the end of the treatment, FAM-VAD-fmk was added as a caspase substrate that exhibits fluorescence after cleavage. Cells were incubated for further 60 min, counterstained with propidium iodide (red fluorescence), and intracellular fluorescence intensity was analysed by microscopy. C: vehicle-treated control cells; TBT: 1 *μ*M TBT; TBT + AEVD: pretreated with zAEVD-fmk for 1 h and 1 *μ*M TBT for further 4 h.

The extrinsic pathway upstream the mitochondria is further characterised by use of two genetically modified Jurkat cell lines where one is caspase-8 deficient and the other is FADD adaptor protein deficient. Furthermore, various caspase inhibitors were used to dissect their roles as possible starting point of the apoptotic sequence of events induced by TBT. Firstly, caspase-8 deficient cells exhibit only a slight reduction of apoptosis in all three cell lines when incubated with 1 or 1.5 *μ*M TBT (Figure 2), whereas Fas-ligand-induced apoptosis was completely abolished (not shown). Without caspase-8, the only measurable protection was found for PS externalisation that is reduced by one third (Figure 3). Secondly, FADD deficiency affords an improved protection against the effects caused by TBT exposure, especially at lower concentrations (Figure 2, 1 *μ*M TBT). This is further corroborated by analysis of PS externalisation and the total caspase activity by a fluorescence assay in living cells. The externalisation of PS was reduced in the same order of magnitude as in the caspase-8-deficient cells (Figure 3), but the CaspaTag assay demonstrates high protease activity in TBT-treated wild-type cells as well as in the caspase-8-deficient variant, whereas in FADD-deficient cells, this activity is obviously reduced (Figure 4).

 The importance of caspases for organotin-provoked apoptosis has been investigated by use of several inhibitors. The overall caspase inhibitor zVAD-fmk blocks totally all described effects that normally can be detected after TBT exposure (data not shown). In this study, we used further specific inhibitors of caspases downstream as well as upstream of mitochondria. When caspase-9 and caspase-3 were inhibited as most potent elements of the caspase cascade downstream of the mitochondria, TBT-induced apoptosis is fully prevented in all three cell lines (Figure 2). Preincubation of the Jurkat cells with zLEHD-fmk (caspase-9 inhibitor) and zDEVD-fmk (caspase-3 inhibitor) rescues all viable functions. Nevertheless, a closer look at the western blots revealed often a slight reduction of those elements that were cleaved upstream of the mitochondria, especially BID, even though at slightly higher concentrations of TBT (Figure 2, 1.5 *μ*M). While caspase-8 is activated in fact after TBT treatment of wild-type Jurkat cells, caspase-8 deficient cells undergo apoptosis to a comparable extent. This result suggests that caspase-8 cannot play a substantial role within this concert of effects after TBT treatment. Therefore, we looked closer for caspase-10, the second initiator caspase at the receptor level.

### 2.3. Caspase-10 Is Obligatory for TBT-Induced Apoptosis, and Its Inhibition Prevents Apoptosis

**Figure 5:**
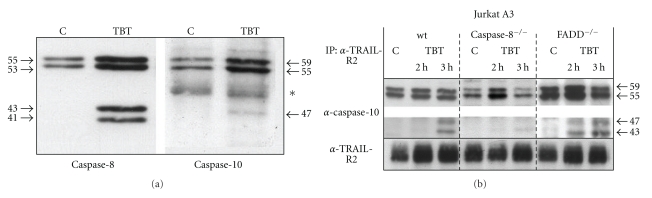
TBT induces recruitment of caspase-8 and caspase-10 by TRAIL-R2. Jurkat A3 wt cells were treated with or without TBT for 3 h before cells were lysed and immunoprecipitation (IP) was carried out with anti-TRAIL-R2 antibody. Initiator caspase-8 and caspase-10 were detected by western blotting (WB) in the precipitates (a). Both antibodies recognise full length procaspases and the processed subunits. Asterisk indicates an unspecific band. Caspase-10 was further investigated in all three cell lines after IP with anti-TRAIL-R2 antibody (b). After 2 or 3 h of incubation with TBT, cells were analysed by IP/WB. In all three cell lines, the amount of procaspase-10 and/or its cleavage products increase over time in the precipitate. Loading control for TRAIL-R2 is shown below. Arrows and numbers give the molecular weight of procaspases and cleaved subunits. Both parts of the western blot (upper part containing the procaspase-10, lower part with the cleavage products) are differentially exposed to visualize the weak bands of the cleavage products.

When caspase-10 is inhibited by zAEVD-fmk, PS externalisation (Figure 3) and overall caspase activity is drastically reduced in all cell lines investigated in this study (Figure 4). Next, we wanted to know if initiator caspases could be found in an activated DISC and which ones. Immunoprecipitations (IP) with an antibody against the Fas-receptor coprecipitated caspase-8 (data not shown), but this caspase has no substantial relevance for the TBT effect in Jurkat cells as shown above. Therefore, we tested Jurkat cells for other death receptors and found additionally TRAIL-R1, TRAIL-R2, TRAIL-R3, TRAIL-R4, and TNF-R1. As TRAIL-R2 was the dominant form and TRAIL-R3 and TRAIL-R4 are decoy receptors, we used a TRAIL-R2 antibody for our IPs. With this antibody, we could precipitate both initiator caspases after 3 h of treatment with 1 *μ*M TBT (Figure 5(a)). Additionally, we detected the procaspases-8 and -10 in the untreated controls but to a much lesser extent and we never found the activated subunits. Analysing the three different cell lines reveals the fact that caspase-10 could be found in all activated DISC forms even though at different levels (Figure 5(b)).

### 2.4. Different Roles of Caspase-8 and Caspase-10 in Fas-Ligand and TBT-Induced Apoptosis

**Figure 6:**
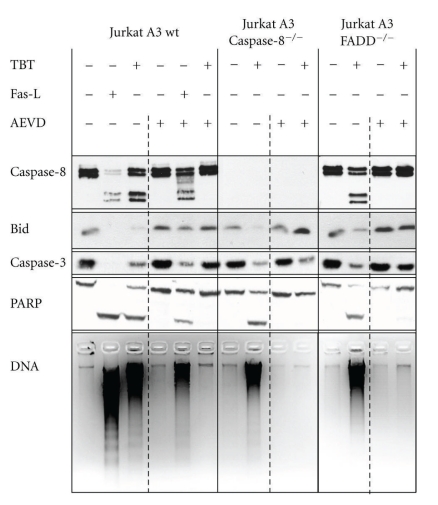
Caspase-10 inhibition prevents all TBT-induced apoptotic effects in Jurkat A3 cells. Jurkat A3 parental and the two deficient cell lines were analysed as shown in Figure 2 except the pretreatment was accomplished with zAEVD-fmk. Jurkat A3 wt cells were treated with 1 *μ*M TBT or Fas-ligand (Fas-L) as positive control for 4 h, and the deficient cell lines were treated with TBT only before proteins were separated on SDS-gels and immunoblotted. A second set of samples was preincubated for 1 h with the caspase-10 inhibitor AEVD (8.7 *μ*M) before TBT or Fas-L were added. Additionally, DNA fragmentation was recorded from the same samples after agarose gel electrophoresis.

A direct comparison of the effects of Fas-ligand and TBT in all three cell lines pre-treated with and without the caspase-10 inhibitor zAEVD-fmk provides a detailed insight into the different roles of the two initiator caspases in human Jurkat T cells. Fas-ligand treatment of the two deficient cell lines has no effect at all, and, thus, these data were not included in Figure 6. The Jurkat A3 wild-type cells, however, were driven into apoptosis, and this effect is only to a minor degree diminished by the pretreatment with the caspase-10 inhibitor, and apoptosis still proceeds. TBT treatment, however, has approximately the same effect as Fas-ligand in the absence of zAEVD-fmk, but all consequences of this treatment were prevented in the presence of AEVD. Phosphatidylserine externalisation is reduced to nearly control levels (Figure 3), and activation of caspases is strongly decreased in all three cell lines (Figure 4). In addition, the cleavage of important caspases is prevented (caspase-8 and caspase-3, Figure 6), BID cleavage is drastically diminished, PARP is completely rescued, and DNA fragmentation does not proceed anymore (Figure 6).

## 3. Discussion

Trialkylated tin compounds, especially TBT, are distributed all over the environment, and were taken up by cells *in vitro* fast and effectively and their toxicity is a function of both concentration and duration of exposure [30]. It has long been discussed that this cytotoxicity of organotin compounds might be the result of a massive alteration of the intracellular calcium concentration [Ca^2+^]_*i*_. Various investigations demonstrated an increase of [Ca^2+^]_*i*_ after exposure to a variety of trialkytins, and this effect should be responsible for their cytotoxicity, immunotoxicity, and neurotoxicity not only in mammalian [14, 17, 30–32] but also in fish cell systems [22]. But more and more evidence has been supplied that alteration of [Ca^2+^]_*i*_ is not the major event in the nonacute cytotoxic scenario [43, 44]. Numerous studies have been carried out during the last two decades indicating the induction of apoptosis in various biological systems without elucidating the starting point of the involved molecular mechanism [14, 17–25, 27, 28, 36]. As early as in 2001, the first publication demonstrated a possible involvement of the death receptors [38], and this was confirmed a few years later [37]. Nevertheless, recently published data connect developmental abnormalities of fish larvae with the induction of apoptosis on the level of caspase 3 [45], and the initiating molecular mechanism by which TBT induces apoptosis is not described. Thus, this study was carried out to enlighten the mechanism in more detail.

There exist two different pathways for apoptosis that can be distinguished from each other, the extrinsic and the intrinsic pathway [46]. The intrinsic pathway is dependent from proapoptotic events on the level of the mitochondria and is mostly affected by environmental chemicals or stress factors. Thus, it seems to be obvious that toxic substances such as TBT exert their effect on mitochondria. A multitude of studies have shown that different parameters of mitochondria were altered after treatment of cells with TBT [18, 32, 37]. Nonetheless, the induction of apoptosis could not be explained sufficiently by all these examinations because mitochondria-independent apoptosis has been described as well [37, 47] and inhibition of the intrinsic pathway by bcl-2 overexpression protects only type II cells but not type I cells from apoptosis although the mitochondrial membrane potential ΔΨ_*m*_ is still high [48]. Moreover, it has been published earlier that various metal compounds may activate the extrinsic apoptotic pathway [37–39, 49]. A closer look on the formation of the “death-inducing signalling complex” (DISC) reveals its formation within 1 to 3 h after treatment with TBT (Figure 5). Normally, in Jurkat T cells, the DISC consists out of the Fas-receptor molecules to which the adaptor molecules FADD and initiator caspase-8 are bound. But the caspase-8-deficient Jurkat cells showed no or only little reduction in apoptosis after TBT treatment, and solely FADD deficiency decreases substantially the apoptotic cell number although not all. So we looked for other elements as possible constituents of the DISC. As it was published by several groups that not only caspase-8 but also caspase-10 can be recruited to death receptors [50, 51], Apo2L/TRAIL is able to activate both initiator caspases, and caspase-10 is described as important caspase in HCT 116 colon carcinoma cells [52], we analysed the DISC formation in more detail. As mentioned above, caspase-10 could be found in the DISC and is co-precipitated by anti-TRAIL-R2 antibody. These results were confirmed by measuring various caspase activities in lysates using the substrates IETD-pNA (caspase-8), AEVD-pNA (caspase-10), and DEVD-pNA (caspase-3), respectively. In lysates of TBT-treated cells, all caspases have been found to be active (data not shown). Thus, TBT leads not only to unspecific cleavage of caspases but directly to their activation.

Because caspase-8-deficient Jurkat cells express lower amounts of caspase-10 compared to their parental cell line, these cells might be somewhat less sensitive to TBT as demonstrated here (Figure 3). But determination of all apoptotic markers revealed a nearly unchanged sensitivity to TBT of Jurkat cells lacking caspase-8 when treatment is prolonged to a minimum of 4 h. Another set of experiments focuses on caspases in more detail. Overall inhibition of caspases with zVAD-fmk, an unspecific inhibitor of all cellular caspases, inhibits totally TBT-induced apoptosis in human neutrophils [19] as well as in Jurkat cells (data not shown). A strong evidence for a specific role of the initiator caspase-10 comes from our experiments with its specific inhibitor zAEVD-fmk. While Fas-ligand-induced apoptosis is only slightly prevented after pre-incubation of the cells with AEVD (Figure 6), TBT-treatment has no effect at all, when caspase-10 was inactivated before. Nevertheless, Kischkel and coworkers [50] described FADD as an obligatory adaptor for both initiator caspases to TRAIL receptor; thus, we expected the FADD-deficient cells to be protected against TBT-induced apoptosis. But this is the case only for lower concentrations of TBT up to 1 *μ*M. In this case, the prevention of FADD-deficient cells is apparent (Figure 2), whereas slightly higher concentrations (1.5 *μ*M) overcome this protective effect. This might be due to the fact that these higher concentrations directly affect, on the one hand, the intrinsic machinery of apoptosis or, on the other hand, TBT might be able to activate directly caspases as has been demonstrated earlier [36, 47]. Another evidence for caspase-10 dependency with no or only less involvement of FADD-adaptor protein has been described recently for another chemical but with the same set of Jurkat cells [53]. This group found the same total inhibition of all effects by the caspase-10 inhibitor zAEVD-fmk and no reduction in caspase-8-deficient cells. Moreover, FADD recruitment was not involved because the FADD-deficient Jurkat cells exhibited DNA fragmentation and other signs of apoptosis; thus, these results are obviously congruent with the data presented here.

Furthermore, it has been published lately that caspase-10 may cleave specific substrates, as the proapoptotic protein BID, without being cleaved before into its active subunits [54]. This may be the reason why type II cells are more sensitive to bcl-2 overexpression than type I cells, as type II cells are dependent on BID cleavage and the activation of the mitochondrial pathway. In our hands, the type I cell line SKW has a higher level of caspase-10 expression, and this is cleaved at the DISC in both variants, the wild type as well as in the bcl-2 overexpressing line (data not shown).

**Figure 7:**
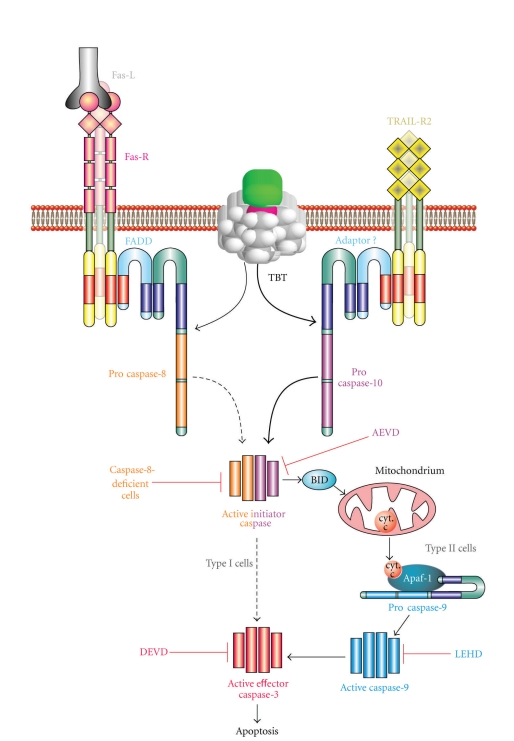
Proposed scheme of TBT-induced apoptosis in Jurkat cells. TBT activates caspase-10 upstream of mitochondria which leads to BID cleavage, and activation of the mitochondria. Downstream caspases were then cleaved and the caspase-cascade is provoked. Caspase-8 deficiency cannot prevent apoptosis, but inhibition of caspase-10 (AEVD) or inhibition of the two important caspases downstream of the mitochondria, caspase-9 and caspase-3 (LEHD and DEVD), totally suppress cell death.

The question to what extent the extrinsic or the intrinsic pathway is responsible for the TBT-induced apoptosis in the absence of a functional FADD adaptor protein can be answered by the concentrations of TBT used within the experiments. Lower concentrations not disturbing the lysosomal or mitochondrial systems are more or less totally dependent on the formation of an activated DISC, whereas higher concentrations overcome this mechanism and stress the intracellular machinery via lysosomes or/and mitochondria leading to caspase-independent responses [55–57]. The here described results indicate that TBT in principle activates initiator caspase-10 leading to BID cleavage and activation of the mitochondria inducing the downstream apoptotic machinery (Figure 7).

 Besides mammalian system cells from other species were affected by TBT as well. In trout blood cells, 1–5 *μ*M TBT induces apoptosis within 1 h [35], and, in gill tissue of the mussel *Mytilus galloprovincialis* treated with 1 *μ*g/g bw TBT (*≈*3 *μ*M), apoptosis could be detected after 24 h incubation [28]. The strongly discussed immunosuppressive properties of TBT *in vivo* might be the consequence of specific induction of cell death in immunocompetent cells. Such a killing of lymphocytes by TBT can be observed as a loss in thymus weight or thymus atrophy [29, 58] that debilitates the immune function of animals, making them vulnerable to infectious diseases [59–63].

On the background of these findings, the deadline for banning TBT must be possibly reconsidered as not only sea snails but also open water mammals and humans might be affected and new regulatory strategies have to be discussed independent from market forces [64]. Whereas the International Maritime Organisation (IMO) has banned TBT since 2008, the European Commission has forbidden its use “after 1st of July 2010 in articles where the concentration in the article, or part thereof, is greater than the equivalent of 0.1% by weight of tin,” but “articles treated with such biocides may still be imported into the Community” [65].

## 4. Materials and Methods

### 4.1. Materials

All cell culture reagents were purchased from Life Technologies (Eggenstein, Germany), petri dishes and multiwell plates were obtained from Nunc (Wiesbaden, Germany). Annexin-FITC is from BD Pharmingen (Heidelberg, Germany), PI and Hoechst 33342 from Sigma (Deisenhofen, Germany), and the inhibitors of caspase-10 (zAEVD-fmk; FMK009), caspase-9 (zLEHD-fmk; FMK008), and caspase-3 (zDEVD-fmk; FMK004) were from R&D Systems (Wiesbaden, Germany).

The primary antibodies anti-caspase-6 (Cat no. 9762), anti-caspase-7 (Cat no. 9492) and anti-caspase-9 (Cat no. 9502) were purchased from Cell Signaling Technology (Frankfurt, Germany), anti-caspase-3 (Cat no. C31720), and anti-caspase-8 (Cat no. 551242 clone 3-1-9) from BD Pharmingen (Heidelberg, Germany), anti-caspase-10/a (Cat no. M059-3) from MoBiTec (MBL) (Göttingen, Germany), anti-TRAIL-R2 (DR5) (Cat no. PC392) from Calbiochem (Darmstadt, Germany), anti-BID (Cat no. AF846) from R&D Systems (Wiesbaden, Germany), and anti-PARP (Cat no. 1835238) from Roche Biochemica (Mannheim, Germany). As secondary reagents, we used: horseradish-peroxidase- (HRP-) conjugated goat anti-mouse IgG1 (Cat no. P 0447) from DakoCytomation (Glostrup, Denmark) and HRP-conjugated donkey anti-rabbit (Cat no. NA934) from Amersham Biosciences (Freiburg, Germany).

### 4.2. Cell Cultures

The Jurkat cell line A3 as well as the FADD and caspase-8-deficient cell lines were kindly provided by J. Blenis (Harvard Medical School, Boston, USA) and were maintained in RPMI 1640 supplemented with 10% FCS and 1 mM HEPES. The cultures were grown with 100 U/mL penicillin and 100 *μ*g/mL streptomycin in a humidified atmosphere containing 5% CO_2_ at 37°C. During experiments, 10 mM glucose were additionally included in the incubation medium.

### 4.3. Methods

#### 4.3.1. Treatment of the Cells

For induction of apoptosis, 2 × 10^6^ cells per mL medium were incubated with 1 *μ*L/mL of a 1 mM stock solution of TBT (Merck) in ethanol to give the final concentration of 1 *μ*M. As a positive control, 100 ng Fas-ligand plus 1 *μ*g enhancer (Alexis, San Diego, USA) per mL incubation medium were used. All controls were incubated with the same amount of vehicle (1 *μ*L ethanol/mL) to exclude side effects of the solvent.

#### 4.3.2. DNA Fragmentation

Apoptotic DNA fragments were isolated according to the following procedure. 2 × 10^6^ were disrupted in 500 *μ*L lysis buffer (20 mM EDTA, 1% NP 40, 50 mM Tris/HCl, pH 7.5). After centrifugation at 1600 g for 5 min, supernatants containing the apoptotic DNA were transferred into Eppendorf tubes. After addition of 1% SDS, samples were treated for 2 h with RNAse-A (5 *μ*g/*μ*L) at 56°C and subsequently for further 2 h with proteinase K (2.5 *μ*g/*μ*L). The DNA was precipitated by the addition of 50 *μ*L of 10 M ammoniumacetate and 250 *μ*L ice-cold ethanol, stored overnight at −20°C followed by centrifugation. The pellet was resuspended in 25 *μ*L of TE-buffer (10 mM Tris/HCl, 1 mM EDTA, pH 8.0), and the DNA-fragments were analysed by agarose gel electrophoresis (1.8% agarose containing ethidium bromide as DNA stain), and pictures were taken with a MWG Gel documentation system.

#### 4.3.3. Caspa-Tag Assay

The assay was carried out as described by the manufacturer (Intergen, Heidelberg, Germany). From each treated sample, 150 *μ*L containing 3 × 10^5^ cells were washed with fresh medium and 10 *μ*L of 30x solution of the caspase substrate FAM-VAD-fmk were added to give the final concentration of 10 *μ*M. The mixture was further incubated for 1 h in an incubator at 37°C. Then, 1 mL of washing solution was added, the suspension was centrifuged at 400 g for 5 min at room temperature. The resulting cell pellet was washed once with washing buffer and finally resuspended in 400 *μ*L washing buffer including 1 *μ*L PI as counterstain for necrosis. Cells were left for 15 min on ice, and then the microscopic pictures were taken.

#### 4.3.4. Immunoprecipitation

TRAIL-associated caspase-10 and caspase-8 were immunoprecipitated as follows: 10^7^ cells (2 × 10^6^/mL) were treated with 1 *μ*M TBT and then lysed in 500 *μ*L cell lysis buffer (30 mM Tris/HCl, pH 7.5, 150 mM NaCl, 1 mM phenylmethylsulfonyl fluoride, 10 *μ*g/mL aprotinin and 10 *μ*g/mL leupeptin, 1% Triton and 10% glycerol). The lysates were centrifuged at 14 000 g for 10 min at 4°C. The supernatants were then incubated for 4 h with protein A-Sepharose (Sigma, Deisenhofen, Germany) and 1.5 *μ*g anti-TRAIL-R2. The beads were centrifuged at 7000 g for 6 min at 4°C, washed once with the same amount of lysis buffer, and centrifuged again. After having removed the supernatants carefully, the pellets were resuspended in 100 *μ*L electrophoresis buffer and 15 *μ*L of each sample were analysed by SDS-gel electrophoresis and western blotting.

#### 4.3.5. Apoptosis Assays

Apoptotic and necrotic cells were determined either microscopically or by flow cytometry using recombinant annexin V conjugated to FITC and propidium iodide. The determination of apoptosis is based on the binding of annexin V-FITC on the phosphatidylserine exposed at the surface of apoptotic cells. Necroses were determined by staining with the membrane impermeable DNA-intercalating dye PI. For the assay, 1 × 10^6^ cells were pelleted at 1500 g and resuspended in 100 *μ*L binding buffer (10 mM HEPES, pH 7.4, 140 mM NaCl and 5 mM CaCl_2_) containing 5 *μ*L annexin V-FITC and 100 ng PI. After 15 min of incubation at 4°C in the dark, cells were diluted in 400 *μ*L binding buffer and immediately analysed by flow cytometry. Fluorescence was analysed at 530 ± 14 nm (FITC) and 610 ± 10 nm (PI) and quantified with the CELLQuest Prosoftware (BD, Heidelberg, Germany).

For microscopic assessment of apoptosis and necrosis, 6 × 10^4^ cells were diluted 1 : 1 with 2x binding buffer, containing 1 *μ*L annexin V-FITC and 3 ng PI. After 15 min of incubation in the dark, cells were analysed with a 63x oil objective and a Zeiss Axiovert S100 microscope (Carl Zeiss GmbH, Jena, Germany), connected to a Hamamatsu CCD Camera (C4880-80). Fluorescence and differential interference contrast pictures were taken using an automation procedure and merged using Openlab software (Improvision, Coventry, UK).

Chromatin condensation was determined after staining of the cells with Hoechst 33342. In short, after treatment, 1 × 10^6^ cells were washed with phosphate-buffered saline (PBS) incubated with a final concentration of 10 *μ*M Hoechst 33342 for 10 min, washed again with PBS to reduce background fluorescence, and finally visualised with the same system described above with excitation at 364 ± 15 nm and emission at 460 ± 10 nm.
